# Attitudes towards snakebite health education and associated factors among residents in China: A national cross-sectional study

**DOI:** 10.7189/jogh.14.04096

**Published:** 2024-05-10

**Authors:** Chuanzhu Lv, Jing Feng, Yanlan Hu, Xingyue Song, Juntao Wang, Wenjie Hao, Lanfen He, Yu Chen, Xiaotong Han, Yong Gan, Shijiao Yan

**Affiliations:** 1 Emergency Medicine Center, Sichuan Provincial People’s Hospital, University of Electronic Science and Technology of China, Chengdu, Sichuan, China; 2 Research Unit of Island Emergency Medicine, Chinese Academy of Medical Sciences (No. 2019RU013), Hainan Medical University, Haikou, Hainan, China; 3 Department of Social Medicine and Health Management, School of Public Health, Tongji Medical College, Huazhong University of Science and Technology, Wuhan, Hubei, China; 4 School of Public Health, Hainan Medical University, Haikou, Hainan, China; 5 Department of Emergency, Hainan Clinical Research Center for Acute and Critical Diseases, The Second Affiliated Hospital of Hainan Medical University, Haikou, Hainan, China; 6 Department of Emergency Medicine, Hunan Provincial Key Laboratory of Emergency and Critical Care Metabolomics, Hunan Provincial Institute of Emergency Medicine, Hunan Provincial People’s Hospital/The First Affiliated Hospital, Hunan Normal University, Changsha, Hunan, China

## Abstract

**Background:**

This study aimed to investigate attitudes towards health education on snakebites and to identify the influencing factors among Chinese residents. Additionally, we proposed effective health education strategies for snakebite management.

**Methods:**

Between May 2022 and February 2023, we conducted a nationwide cross-sectional questionnaire survey using a multistage sampling method with supplementary snowball sampling. We used descriptive analysis, χ^2^ tests, and univariable and multivariable binary logistic regression models to analyse the data.

**Results:**

We included 56 669 respondents in the analysis. The average score for snakebite knowledge was 12.13 ± 5.26 points, with a maximum score of 28. Among the respondents, 72.66 and 63.03% of the residents believed that it was necessary to disseminate knowledge about snakebites and expressed a willingness to receive snakebite training, respectively. Respondents from the northeast region, respondents with a higher education level, and respondents with higher scores for snakebite knowledge, health knowledge, health skills, and social-psychological adjustment skills exhibited more positive attitudes towards snakebite knowledge dissemination and training. Conversely, respondents from eastern or southern China, respondents older than 60, and respondents who lived in rudimentary housing conditions showed a lower perception of the need for snakebite knowledge dissemination and were less willing to participate in snakebite knowledge and skill training.

**Conclusions:**

Generally, Chinese residents have positive attitudes towards snakebite knowledge dissemination and training. However, the public lacks sufficient knowledge about snakebites. Therefore, we should pay close attention to areas south of the Yangtze River to strengthen snakebite health education using engaging formats that align with residents’ interests, such as short videos or television programmes, in an attempt to and ultimately improve health literacy and prevention awareness.

Snakebites are prevalent animal-induced injuries and represent a significant global health concern. More than 5.8 billion individuals worldwide face the risk of encountering venomous snakes, with nearly 7400 snakebite incidents and 220 to 380 fatalities reported each day [1]. Annually, there are approximately 2.7 million cases of snakebite envenomation, resulting in 81 000 to 138 000 deaths [1]. Non-venomous snakebites primarily cause localised injuries, whereas venomous snakebites lead to acute systemic poisoning as venom enters the human body through the wound [2]. Due to the rapid onset and progression of snakebite symptoms, timely and appropriate treatment is crucial. Without prompt intervention, snake venom can rapidly spread within the body, affecting multiple organ functions and leading to metabolic disruptions, multiple organ failure, and potential death [2]. Additionally, venomous snakebites may result in long-term physical consequences, including amputations, paralysis, disabilities, and psychological implications [3–6]. In the majority of countries, the economic burden of snakebites poses challenges to effective management and affects not only victims but also their entire family, particularly in impoverished communities in low- and middle-income countries that lack social security [7]. Given the gravity of this issue, the World Health Organization designated snakebite as a neglected tropical disease (NTD) in 2017, urged global collaboration to control snakebite incidents, and established strategic objectives to reduce snakebite-related mortality and disability by 50% by 2030 [1].

Effectively controlling snakebite incidents requires an improved focus on health education pertaining to risks that includes raising residents’ awareness of snakebites, encouraging timely medical care for affected individuals, and ensuring robust case management for all patients. A systematic review and meta-analysis revealed that only 55% of the general population displayed a satisfactory understanding of snakebites and their management [8]. Specifically, knowledge of antivenom therapy was relatively high, whereas first aid knowledge for snakebites was notably lacking. Consequently, it is imperative to reinforce health education efforts related to snakebite management. Given the influential role of attitudes towards health education in driving active engagement, an exploration of residents’ attitudes towards snakebite health education is instrumental in designing tailored interventions to increase participation rates and improve snakebite prevention and control measures to ultimately alleviate the overall burden of snakebite-related illnesses.

There are several million snakebite cases annually in China, with 100 000 to 300 000 of these incidents involving venomous snakebites and over 70% involving young and middle-aged adults [2]. The fatality rate of snakebites is approximately 5%, and snakebites result in disability for 25 to 30% of affected individuals; this significantly affects the productivity of the labour force and places a substantial burden on families and society [2]. A previous study investigated snakebite knowledge among Chinese field forces [9], but there remains a dearth of research on snakebite knowledge and health education attitudes among the general population. To address this gap, this study assessed the level of snakebite knowledge among Chinese residents, conducted a pioneering analysis of their health education attitudes towards snakebites, and explored the factors that influence these attitudes. The primary objectives were to identify target groups for key educational interventions and to propose scientifically robust health education strategies to enhance public awareness and preventive behaviours related to snakebites. Ultimately, this research will serve as a benchmark for snakebite prevention and control efforts not only in China but also in other countries.

## METHODS

### Study population

We conducted a cross-sectional study in China between May 2022 and February 2023. We used a multistage sampling method with complementary snowball sampling to select the research population. Multistage sampling provided a structured framework for obtaining a representative initial population, while snowball sampling supplemented this population by identifying individuals who may have been missed by conventional sampling methods. Snakebite incidents may be more prevalent in certain regions, and snowball sampling allowed the researchers to reach these populations to improve the representativeness of the study. Based on a comprehensive literature search and pragmatic considerations, we initially selected 12 provinces/municipalities/autonomous regions with a notable prevalence of severe snakebite incidents in the Yangtze River Basin and southern regions. These provinces included Hubei, Hunan, Guangdong, Guangxi, Hainan, Zhejiang, Fujian, Jiangxi, Sichuan, Guizhou, Yunnan, and Chongqing. Subsequently, we used a convenience sampling approach to identify three cities within each selected province/municipality/autonomous region, three districts/counties within each city, and three communities/villages within each district/county. Within each community/village, we chose 30 to 50 residents to participate in the questionnaire. Additionally, to supplement the national survey data and cover areas not initially selected, we disseminated electronic questionnaires through popular platforms such as WeChat and QQ by employing the snowball sampling method.

The Ethics Committee of Hainan Medical College approved the study protocol (No. HYLL-2022-226). We obtained informed consent from all survey participants and the guardians of minors.

### Measurement

#### Dependent variable

We assessed attitudes towards snakebite health education using three specific items. For the question, ‘Do you think it is necessary to carry out knowledge popularisation regarding venomous snakebites?’, we coded the responses as ‘strongly necessary’ (1), ‘necessary’ (1), ‘neutral’ (0), and ‘not necessary’ (0). For the question, ‘Are you willing to participate in training on knowledge and skills for snakebites?’, we coded the responses as ‘willing’ (1), ‘unwilling’ (0), and ‘unsure’ (0). For the question, ‘What forms of snakebite health education would you prefer to receive?’ (multiple-choice question), the responses encompassed five options: ‘short videos’, ‘television programmes’, ‘offline lectures’, ‘books or relevant manuals’, and ‘other’.

#### Independent variables

The independent variables included demographic characteristics (region, sex, age, marital status, education level, occupation, manner of payment for health care expenses, and housing type), personal experiences and surrounding experiences of snakebite incidents, snakebite knowledge, and health literacy.

The snakebite knowledge section consisted of 16 items, comprising four single-choice questions and 12 multiple-choice questions. For single-choice questions, a correct response received 1 point, while incorrect or unknown answers received 0 points. For multiple-choice questions, we assigned 2 points for correct answers, 1 point for partially correct answers, and 0 points for incorrect or unknown responses. The cumulative score ranged from 0 to 28, with higher scores indicating a greater level of snakebite awareness.

We measured health literacy using the Health Literacy Scale developed by Jin [10], which comprised 26 items categorised into five dimensions: health awareness and concepts (six items), health knowledge (seven items), health skills (three items), social-psychological adjustment skills (three items), and health information cognition and application (seven items). The scoring methodology combined the difficulty coefficient approach with a five-level Likert scale [10]. We calculated each item’s score based on its difficulty coefficient, which represented the reciprocal of the correct response rate. Higher difficulty coefficients corresponded to lower correct response rates, resulting in higher scores for correct answers. The scoring formula was as follows: score = n/m, where ‘n’ denoted the total number of survey participants and ‘m’ represented the number of participants who answered the item correctly. For example, if an item had a correct response rate of 10% and a difficulty coefficient of 10, the maximum score for that item would be 10 points. With a correct response rate of 50%, the corresponding score would be 2 points. We averaged the difficulty coefficient scores into five levels, with scores assigned to each option. For instance, we graded an item with a difficulty coefficient of 10 as follows: 10 points for option 1, 7.5 points for option 2, 5 points for option 3, 2.5 points for option 4, and 0 points for option 5. The same scoring principle applied to reverse-scored items. Combining the difficulty coefficient approach with the five-level scoring system not only distinguished scores based on varying levels of participant knowledge but also considered the differing levels of item difficulty to provide a more comprehensive assessment of health literacy. In this study, the Cronbach’s α coefficients for the five dimensions of the health literacy scale, namely, health awareness and concepts, health knowledge, health skills, social-psychological adjustment skills, and health information cognition and application, were 0.91, 0.89, 0.75, 0.90, and 0.86, respectively, indicating good reliability.

### Quality control

We developed the survey questionnaire based on a literature review, expert consultations, and group discussions. In the study design stage, we invited 30 health care experts to evaluate the questionnaire’s content validity. We conducted a pilot study among 90 residents in three cities of Hainan Province. A total of 86 respondents were able to comprehend the entire questionnaire. We collected feedback from respondents to make necessary revisions to the questionnaire. Subsequently, with the support of the Emergency Medicine Branch of the Chinese Medical Association, we collected data using electronic questionnaires designed by Wenjuanxing and a mini programme. To ensure data accuracy and prevent duplicate entries, we restricted each device or account to completing the questionnaire only once.

### Statistical analysis

We used Stata 17.0 to conduct data analysis. We presented quantitative variables using means and standard deviations, and reported categorical variables as frequencies and percentages. We used the χ^2^ tests to assess differences in attitudes towards snakebite knowledge dissemination and willingness to participate in training among the various groups. Additionally, we applied univariable and multivariable logistic regression models to identify factors associated with attitudes towards knowledge about snakebite dissemination and the willingness to participate in snakebite training. A two-tailed P-value less than 0.05 indicated statistical significance.

## RESULTS

Initially, we collected 56 803 questionnaires, and included 56 669 questionnaires in the final analysis after excluding 134 questionnaires with missing values. The respondents lived in 34 provincial administrative regions in China, with the majority of respondents coming from eastern China (31.50%). The respondents were predominantly male (57.56%), between the ages of 18 and 40 years (60.01%), and married (60.01%). Approximately one-fifth of the residents were either illiterate or had completed only primary school. Farmers, health care workers, field workers, and snake breeders accounted for 15.62, 10.38, 1.06, and 0.89% of the sample, respectively. Approximately four-fifths of the respondents had medical insurance, and the majority resided in multi-story buildings (69.89%). Furthermore, 12.31% of the respondents had experienced snakebites, and 28.12% reported knowing someone in their vicinity who had been bitten by a snake. The average score for snakebite knowledge was 12.13 ± 5.26 points, and the health literacy scores for the five dimensions were as follows: health awareness and concepts, 13.99 ± 5.78 points; health knowledge, 16.74 ± 5.74 points; health skills, 7.30 ± 2.97 points; social-psychological adjustment skills, 8.16 ± 3.79 points; and health information cognition and application, 20.50 ± 3.96 points (**Table 1**).

**Table 1:** Descriptive statistics and χ^2^ tests of the differences in attitudes towards snakebite health education among residents

Variables	Frequency (%)	Positive attitudes towards snakebite knowledge popularisation	χ^2^/*t*	Being willing to participate in snakebite training	χ^2^/*t*
Total	56 669 (100.00)	41 178 (72.66)		35 721 (63.03)	
Region			966.42*		1100.00*
*Northern China*	12 531 (22.11)	9142 (72.96)		7702 (61.46)	
*Northeast China*	890 (1.57)	776 (87.19)		675 (75.84)	
*Eastern China*	17 852 (31.50)	11 640 (65.20)		9756 (54.65)	
*Central China*	10 733 (18.94)	8013 (74.66)		7315 (68.15)	
*Southern China*	7080 (12.49)	5444 (76.89)		4928 (69.60)	
*Southwest China*	7371 (13.01)	5980 (81.13)		5179 (70.26)	
*Northwest China*	212 (0.37)	183 (86.32)		166 (78.30)	
Sex			3.45		4.51†
*Male*	32 618 (57.56)	23 799 (72.96)		20 440 (62.66)	
*Female*	24 051 (42.44)	17 379 (72.26)		15 281 (63.54)	
Age in years			1500.00*		971.53*
*<18*	3215 (5.67)	2354 (73.22)		1780 (55.37)	
*18–40*	34 008 (60.01)	26 209 (77.07)		22 680 (66.69)	
*41–60*	13 888 (24.51)	9692 (69.79)		8706 (62.69)	
*>60*	5558 (9.81)	2923 (52.59)		2555 (45.97)	
Marital status			1500.00*		2000.00*
*Married*	34 006 (60.01)	25 255 (74.27)		22 880 (67.28)	
*Unmarried*	17 305 (30.54)	13 157 (76.03)		10 920 (63.10)	
*Divorced*	3737 (6.59)	2129 (56.97)		1439 (38.51)	
*Widowed*	1621 (2.86)	637 (39.30)		482 (29.73)	
Education level			1700.00*		2000.00*
*Illiteracy or primary school*	10 835 (19.12)	6451 (59.54)		5223 (48.20)	
*Middle school/high school/technical secondary school*	23 084 (40.73)	16 387 (70.99)		13 937 (60.38)	
*College and above*	22 750 (40.15)	18 340 (80.62)		16 561 (72.80)	
Occupation			676.64*		750.55*
*Farmers*	8853 (15.62)	5771 (65.19)		5225 (59.02)	
*Health care workers*	5882 (10.38)	4952 (84.19)		4552 (77.39)	
*Field workers*	601 (1.06)	400 (66.56)		295 (49.08)	
*Snake breeders*	504 (0.89)	319 (63.29)		204 (40.48)	
*Others*	40 829 (72.05)	29 736 (72.83)		25 445 (62.32)	
Manner of payment for health care expenses			88.79*		84.87*
*Out-of-pocket payment*	8365 (14.76)	6001 (71.74)		5098 (60.94)	
*Health insurance*	45 793 (80.81)	33 527 (73.21)		29 213 (63.79)	
*Free medical treatment*	2363 (4.17)	1531 (64.79)		1312 (55.52)	
*Others*	148 (0.26)	119 (80.41)		98 (66.22)	
Housing type			678.36*		1800.00*
*Multi-story buildings*	39 607 (69.89)	30 035 (75.83)		27 195 (68.66)	
*Adobe/wood/tiles/thatch/bamboo houses*	14 459 (25.51)	9466 (65.47)		7332 (50.71)	
*Tents*	2393 (4.22)	1520 (63.52)		1066 (44.55)	
*Others*	210 (0.37)	157 (74.76)		128 (60.95)	
Personal experiences of snakebite incidents			491.99*		420.33*
*Yes*	6977 (12.31)	5843 (83.75)		5172 (74.13)	
*No*	49 692 (87.69)	35 335 (71.11)		30 549 (61.48)	
Surrounding experiences of snakebite incidents			3400.00*		3800.00*
*Yes*	15 934 (28.12)	13 636 (85.58)		12 501 (78.45)	
*No*	24 046 (42.43)	18 014 (74.91)		15 588 (64.83)	
*Unsure*	16 689 (29.45)	9528 (57.09)		7632 (45.73)	
Snakebite knowledge	12.13 ± 5.26	13.46 ± 4.37	−95.52*	13.84 ± 4.17	−100.00*
Health awareness and concepts	13.99 ± 5.78	14.46 ± 5.77	−32.39*	14.76 ± 5.70	−42.05*
Health knowledge	16.74 ± 5.74	17.40 ± 5.81	−48.60*	17.76 ± 5.77	−58.69*
Health skills	7.30 ± 2.97	7.57 ± 2.96	−35.81*	7.72 ± 2.94	−45.02*
Social-psychological adjustment skills	8.16 ± 3.79	8.43 ± 3.82	−29.28*	8.62 ± 3.81	−39.44*
Health information cognition and application	20.50 ± 3.96	20.84 ± 4.08	−35.86*	21.06 ± 4.14	−46.38*

*P < 0.001.

†*P* < 0.05.

A total of 72.66% of the residents considered it essential to disseminate knowledge about venomous snakebites, and 63.03% of the residents expressed willingness to participate in training about snakebite knowledge and skills. Regarding the preferred forms of snakebite education, 57.36% favoured short videos, 51.27% preferred television programmes, 37.84% opted for offline lectures, 33.56% were interested in books or relevant handbooks, and 1.95% chose other forms.

We observed statistically significant differences in attitudes towards snakebite knowledge dissemination and training willingness among residents based on their geographical region, age group, marital status, educational background, occupation, manner of payment for health care expenses, housing type, personal and surrounding experiences with snakebites, scores of snakebite knowledge, health awareness and concepts, health knowledge, health skills, social-psychological adjustment skills, and health information cognition and application (**Table 1**).

After controlling for other variables, respondents from the northeast region (odds ratio (OR) = 1.83), with a middle school/high school/technical secondary school education (OR = 1.20) or a college education or higher (OR = 1.33), worked in a health care profession (OR = 1.41), and with higher scores for snakebite knowledge (OR = 1.16), health knowledge (OR = 1.07), health skills (OR = 1.10), and social-psychological adjustment skills (OR = 1.01) were more likely to perceive a greater need to promote snakebite knowledge dissemination. Conversely, respondents from eastern (OR = 0.54) or southern China (OR = 0.85), females (OR = 0.92), respondents who resided in houses made of adobe, wood, tiles, thatch, bamboo (OR = 0.86) or tents (OR = 0.71), and those with higher scores for health awareness and concepts (OR = 0.99) were less likely to perceive the necessity of promoting snakebite knowledge dissemination (**Table 2**).

**Table 2:** The univariable and multivariable logistic regression models of the attitudes towards snakebite knowledge popularisation

Variables	Unadjusted model*			Adjusted model†		
	**OR**	**95% CI**	***P-*value**	**OR**	**95% CI**	***P-*value**
Region (ref: northern China)						
*Northeast China*	2.52	2.06–3.08	<0.001	1.83	1.46–2.29	<0.001
*Eastern China*	0.69	0.66–0.73	<0.001	0.54	0.50–0.57	<0.001
*Central China*	1.09	1.03–1.16	0.003	1.07	0.99–1.15	0.086
*Southern China*	1.23	1.15–1.32	<0.001	0.85	0.78–0.92	<0.001
*Southwest China*	1.59	1.49–1.71	<0.001	0.96	0.88–1.04	0.322
*Northwest China*	2.34	1.58–3.47	<0.001	1.36	0.87–2.13	0.177
Sex (ref: male)						
*Female*	0.97	0.93–1.00	0.063	0.92	0.88–0.97	<0.001
Age in years (ref: <18)						
*18–40*	1.23	1.13–1.33	<0.001	0.81	0.73–0.89	<0.001
*41–60*	0.84	0.78–0.92	<0.001	0.65	0.59–0.72	<0.001
*>60*	0.41	0.37–0.45	<0.001	0.51	0.45–0.57	<0.001
Marital status (ref: married)						
*Unmarried*	1.10	1.05–1.15	<0.001	0.96	0.90–1.01	0.129
*Divorced*	0.46	0.43–0.49	<0.001	0.53	0.49–0.57	<0.001
*Widowed*	0.22	0.20–0.25	<0.001	0.41	0.37–0.46	<0.001
Education level (ref: illiteracy or primary school)						
*Middle school/high school/technical secondary school*	1.66	1.59–1.74	<0.001	1.20	1.13–1.27	<0.001
*College and above*	2.83	2.69–2.97	<0.001	1.33	1.24–1.43	<0.001
Occupation (ref: farmers)						
*Health care workers*	2.84	2.62–3.09	<0.001	1.41	1.27–1.56	<0.001
*Field workers*	1.06	0.89–1.27	0.495	0.97	0.79–1.19	0.780
*Snake breeders*	0.92	0.76–1.11	0.386	0.93	0.75–1.14	0.475
*Others*	1.43	1.36–1.50	<0.001	1.15	1.07–1.22	<0.001
Manner of payment for health care expenses (ref: out-of-pocket payment)						
*Health insurance*	1.08	1.02–1.13	0.005	0.93	0.88–0.99	0.028
*Free medical treatment*	0.72	0.66–0.80	<0.001	0.88	0.78–0.99	0.030
*Others*	1.62	1.07–2.43	0.021	1.13	0.68–1.85	0.641
Housing type (ref: multi-story buildings)						
*Adobe/wood/tiles/thatch/bamboo houses*	0.60	0.58–0.63	<0.001	0.86	0.82–0.91	<0.001
*Tents*	0.55	0.51–0.60	<0.001	0.71	0.64–0.79	<0.001
*Others*	0.94	0.69–1.29	0.718	1.06	0.74–1.52	0.761
Personal experiences of snakebite incidents (ref: no)						
*Yes*	2.09	1.96–2.24	<0.001	1.59	1.47–1.72	<0.001
Surrounding experiences of snakebite incidents (ref: no)						
*Yes*	1.99	1.88–2.09	<0.001	1.83	1.73–1.94	<0.001
*Unsure*	0.45	0.43–0.46	<0.001	0.81	0.77–0.85	<0.001
Snakebite knowledge	1.20	1.19–1.20	<0.001	1.16	1.16–1.17	<0.001
Health awareness and concepts	1.05	1.05–1.06	<0.001	0.99	0.98–0.99	0.001
Health knowledge	1.08	1.07–1.08	<0.001	1.07	1.07–1.08	<0.001
Health skills	1.12	1.11–1.12	<0.001	1.10	1.09–1.11	<0.001
Social-psychological adjustment skills	1.07	1.07–1.08	<0.001	1.01	1.01–1.02	0.001
Health information cognition and application	1.09	1.08–1.09	<0.001	1.00	0.99–1.01	0.724
Constant				0.13	0.11–0.15	<0.001

CI – confidence interval, OR – odds ratio

*There was just one explanatory variable in the model.

†The model was adjusted for region, sex, age, marital status, education level, occupation, manner of payment for health care expenses, housing type, personal experiences of snakebite incidents, surrounding experiences of snakebite incidents, snakebite knowledge, health awareness and concepts, health knowledge, health skills, social-psychological adjustment skills, and health information cognition and application. LR chi2(34) = 14501.95, *P* < 0.001.

After controlling for other variables, respondents from the northeast (OR = 1.24) or central China (OR = 1.17), with a middle school/high school/technical secondary school education (OR = 1.27) or a college education or higher (OR = 1.41), worked in the health care field (OR = 1.13), and with higher scores in snakebite knowledge (OR = 1.16), health knowledge (OR = 1.06), health skills (OR = 1.11), social-psychological adjustment skills (OR = 1.03), and health information cognition and application (OR = 1.02) were more willing to participate in snakebite knowledge and skills training. Nevertheless, respondents from eastern China (OR = 0.52), southern China (OR = 0.82), and southwest China (OR = 0.74), field workers (OR = 0.67) or snake breeders (OR = 0.50), and respondents who resided in adobe, wooden, tile, thatch, or bamboo houses (OR = 0.68) or tents (OR = 0.53) were less willing to participate in snakebite knowledge and skills training (**Table 3**).

**Table 3:** The univariable and multivariable logistic regression models of the willingness to participate in snakebite training

Variables	Unadjusted model*			Adjusted model†		
	**OR**	**95% CI**	***P-*value**	**OR**	**95% CI**	***P-*value**
Region (ref: northern China)						
*Northeast China*	1.97	1.68–2.30	<0.001	1.24	1.03–1.50	0.025
*Eastern China*	0.76	0.72–0.79	<0.001	0.52	0.49–0.55	<0.001
*Central China*	1.34	1.27–1.42	<0.001	1.17	1.09–1.26	<0.001
*Southern China*	1.44	1.35–1.53	<0.001	0.82	0.76–0.89	<0.001
*Southwest China*	1.48	1.39–1.58	<0.001	0.74	0.69–0.80	<0.001
*Northwest China*	2.26	1.63–3.14	<0.001	1.22	0.83–1.80	0.320
Sex (ref: male)						
*Female*	1.04	1.00–1.07	0.034	0.97	0.93–1.01	0.110
Age in years (ref: <18)						
*18–40*	1.61	1.50–1.74	<0.001	1.04	0.95–1.13	0.400
*41–60*	1.35	1.25–1.46	<0.001	0.97	0.88–1.07	0.523
*>60*	0.69	0.63–0.75	<0.001	0.79	0.71–0.88	<0.001
Marital status (ref: married)						
*Unmarried*	0.83	0.80–0.86	<0.001	0.81	0.77–0.85	<0.001
*Divorced*	0.30	0.28–0.33	<0.001	0.39	0.36–0.42	<0.001
*Widowed*	0.21	0.18–0.23	<0.001	0.43	0.38–0.48	<0.001
Education level (ref: illiteracy or primary school)						
*Middle school/high school/technical secondary school*	1.64	1.56–1.71	<0.001	1.27	1.20–1.35	<0.001
*College and above*	2.88	2.74–3.02	<0.001	1.41	1.32–1.50	<0.001
Occupation (ref: farmers)						
*Health care workers*	2.38	2.21–2.56	<0.001	1.13	1.03–1.25	0.011
*Field workers*	0.67	0.57–0.79	<0.001	0.67	0.55–0.81	<0.001
*Snake breeders*	0.47	0.39–0.57	<0.001	0.50	0.41–0.62	<0.001
*Others*	1.15	1.10–1.20	<0.001	0.93	0.87–0.99	0.024
Manner of payment for health care expenses (ref: out-of-pocket payment)						
*Health insurance*	1.13	1.08–1.18	<0.001	0.98	0.93–1.04	0.530
*Free medical treatment*	0.80	0.73–0.88	<0.001	1.04	0.93–1.17	0.491
*Others*	1.26	0.89–1.77	0.193	0.86	0.55–1.34	0.497
Housing type (ref: multi-story buildings)						
*Adobe/wood/tiles/thatch/bamboo houses*	0.47	0.45–0.49	<0.001	0.68	0.65–0.72	<0.001
*Tents*	0.37	0.34–0.40	<0.001	0.53	0.48–0.58	<0.001
*Others*	0.71	0.54–0.94	0.017	0.83	0.60–1.16	0.280
Personal experiences of snakebite incidents (ref: no)						
*Yes*	1.80	1.70–1.90	<0.001	1.75	1.63–1.87	<0.001
Surrounding experiences of snakebite incidents (ref: no)						
*Yes*	1.98	1.89–2.07	<0.001	1.92	1.82–2.03	<0.001
*Unsure*	0.46	0.44–0.48	<0.001	0.74	0.70–0.78	<0.001
Snakebite knowledge	1.21	1.21–1.22	<0.001	1.16	1.15–1.17	<0.001
Health awareness and concepts	1.06	1.06–1.07	<0.001	1.00	1.00–1.01	0.373
Health knowledge	1.09	1.09–1.09	<0.001	1.06	1.05–1.07	<0.001
Health skills	1.14	1.13–1.15	<0.001	1.11	1.10–1.12	<0.001
Social-psychological adjustment skills	1.09	1.09–1.10	<0.001	1.03	1.02–1.04	<0.001
Health information cognition and application	1.11	1.11–1.12	<0.001	1.02	1.01–1.02	<0.001
Constant				0.04	0.03–0.05	<0.001

CI – confidence interval, OR – odds ratio

*There was just one explanatory variable in the model.

†The model was adjusted for region, sex, age, marital status, education level, occupation, manner of payment for health care expenses, housing type, personal experiences of snakebite incidents, surrounding experiences of snakebite incidents, snakebite knowledge, health awareness and concepts, health knowledge, health skills, social-psychological adjustment skills, and health information cognition and application. LR chi2(34) = 17 909.58, *P* < 0.001.

## DISCUSSION

Regular and systematic health education plays a crucial role in mitigating the risk of snakebite occurrence and fostering health care-seeking behaviour among snakebite victims, which significantly contributes to snakebite prevention and control [7]. This study found that 72.66% of residents acknowledged the need to promote knowledge about snakebites and 63.03% of residents were willing to participate in training on snakebite skills, indicating a positive attitude towards snakebite health education among the majority of the public. A study in southeast China showed that 86.4% of soldiers exhibited a high demand for snakebite-related knowledge [9]. A pronounced inclination towards health education usually implies higher levels of engagement and compliance, which are advantageous for the effective implementation of health education initiatives.

More than half of the residents expressed a preference for receiving snakebite knowledge education through short videos or television, with short videos being the most preferred option. Short videos are primarily shared through social media, which can effectively address physical barriers to health care support and resources and mitigate issues related to health care disparities [11,12]. As an influential tool for patient education, short videos are crucial for delivering relevant information. However, in the context of an abundance of false information and fragmented knowledge dissemination coupled with a vast array of media choices, the public may be overwhelmed by the sheer volume of content [11]. Thus, the dissemination of authentic and reliable educational materials becomes paramount. Conversely, only approximately one-third of the residents expressed interest in attending offline lectures. Traditional health promotion lectures offer unique experiential learning opportunities that are difficult to replicate through media-based approaches. In the current age of extensive social media usage, there is a need to reinvent and adapt traditional lecture methodologies by shifting the focus from solely disseminating knowledge to encouraging the public’s proactive engagement in seeking health-related information [13]. By incorporating elements of entertainment and appeal, offline lectures can be enhanced to strike a balance between entertainment, science communication, and practicality.

Residents with higher scores for snakebite knowledge exhibited a more positive attitude towards snakebite knowledge dissemination and a greater willingness to participate in training. Those with lower levels of snakebite awareness were prone to underestimate the risk of snakebite exposure and the potentially severe consequences [8]. This led them to perceive snakebite knowledge dissemination as unnecessary and made them reluctant to engage in related training. Moreover, due to the cross-sectional nature of this study, we were unable to establish causal relationships. It was plausible that residents with poorer attitudes towards snakebite health education might not demonstrate sufficient attention to relevant knowledge or active participation in educational activities, resulting in lower knowledge scores. Notably, in this study, residents’ average snakebite knowledge score was 12.13 ± 5.26 points, which fell below the theoretical average of 14 points and indicated a deficiency in snakebite knowledge. Previous studies found that there was inadequate knowledge about snakebites among the general population in India [14] and Myanmar [15], which were similar to China in terms of agriculture-dependent rural economies, tropical climates, and the presence of venomous snakes. It needs further health education for the public. Furthermore, residents with lower educational attainment demonstrated less proactive attitudes towards health education, suggesting that limited educational opportunities hindered awareness of the importance of seeking health-related information. At the community level in China, snakebite health education is not compulsory, and educational resources are scarce. Given the substantial disease burden associated with snakebite and the prevailing knowledge gaps among Chinese residents, we recommend conducting appropriate snakebite health education initiatives within the community. By employing easily understandable language, these initiatives can effectively disseminate fundamental snake characteristics as well as snakebite prevention and first aid knowledge.

The positive association between health knowledge and residents’ attitudes towards snakebite knowledge dissemination and training further underscored the importance of individual cognition in health education. Moreover, both health skills and social-psychological adjustment skills positively predicted residents’ attitudes towards snakebite knowledge dissemination and their willingness to participate in training. However, the dimension of health awareness and concepts exhibited a weak negative predictive effect on residents’ attitudes towards snakebite knowledge dissemination, and its impact on training willingness was not statistically significant. Additionally, the dimension of health information cognition and application was not significantly associated with residents’ attitudes towards snakebite knowledge dissemination. One possible attribution was the prevailing negligence towards snakebites in recent years [16], which restricted the impact of health awareness and concepts, as well as health information cognition and application in shaping attitudes towards snakebite health education.

The results of the multivariable logistic regression analysis revealed that personal or surrounding experiences of snakebite significantly influenced residents’ attitudes towards snakebite health education. Personal experiences underlie attitude formation [17], and the influence of others’ experiences through observation or word of mouth can impact individuals’ perceptions. Compared to residents in northern China, residents in eastern China and southern China demonstrated a lower perceived need for knowledge about snakebite dissemination and a lower willingness to participate in snakebite training, with the lowest percentages found in eastern China at 65.20 and 54.65%, respectively. It is important to note that the eastern China and southern China regions are predominantly situated south of the Yangtze River, where venomous snakes are primarily distributed [18]. Consequently, we should pay attention to the prevalence of snakebites in high-risk areas such as eastern China and southern China, and implement proactive measures to enhance residents’ awareness of snakebite prevention and control in these high-risk areas. Additionally, living in rudimentary housing conditions had a negative predictive effect on residents’ attitudes towards snakebite health education. As these living environments are often in proximity to snake habitats [1], it is crucial to implement targeted prevention measures that respect the ecological environment, such as sleeping on a raised bed [19].

In contrast to previous studies that predominantly focused on the diagnosis and treatment of snakebites [20], we focused on prevention and control by assessing Chinese residents’ attitudes towards the dissemination of knowledge about snakebites. This study provided a baseline overview of attitudes towards snakebite health education among the Chinese public. The results confirmed that Chinese residents have a positive attitude towards snakebite health education and that improving the dissemination of snakebite knowledge and health literacy is helpful for the prevention and control of snakebite. Our findings cannot only provide evidence for health education strategies about snakebites among residents in China but also serve as a reference for international comparisons.

### Strengths and limitations

This nationwide, large-scale, cross-sectional survey is the first to explore residents’ attitudes towards the dissemination of knowledge about snakebites and their willingness to participate in related training. This study therefore offers valuable insights for snakebite health education in China and other regions with similar snakebite issues. However, we acknowledged that there were certain limitations in the study. First, the large sample size may have magnified slight differences, although we adjusted for potential confounding variables in the multivariable logistic regression model. Second, other factors may be associated with residents’ attitudes towards snakebite health education, such as lifestyle behaviours and residential environments, which we did not investigate in this study. Third, the use of electronic questionnaires may have led to response bias. Despite our efforts to ensure anonymity and offer clear instructions, participants might be inclined to provide socially desirable responses or might not take the survey seriously, potentially resulting in data inaccuracies. In the future, we should take targeted interventions to improve the snakebite knowledge and prevention behaviours of high-risk residents.

## CONCLUSIONS

Chinese residents show a predominantly positive attitude towards the dissemination of snakebite knowledge and willingness to engage in relevant training. However, lower levels of education, modest housing conditions, and limited awareness of snakebite-related information are the main barriers to snakebite health education. Improving health literacy in areas such as health knowledge, health skills, and social-psychological adjustment skills may improve residents’ attitudes towards snakebite health education. It is suggested to focus on priority regions south of the Yangtze River and employ preferred educational methods, such as short videos or television, to enhance the dissemination of snakebite knowledge and ultimately alleviate the burden of snakebite-related diseases.
